# Noise in Attractor Networks in the Brain Produced by Graded Firing Rate Representations

**DOI:** 10.1371/journal.pone.0023630

**Published:** 2011-09-08

**Authors:** Tristan J. Webb, Edmund T. Rolls, Gustavo Deco, Jianfeng Feng

**Affiliations:** 1 Department of Computer Science and Complexity Science Centre, University of Warwick, Coventry, United Kingdom; 2 Oxford Centre for Computational Neuroscience, Oxford, United Kingdom; 3 Department of Computer Science, University of Warwick, Coventry, United Kingdom; 4 Theoretical and Computational Neuroscience, Universitat Pompeu Fabra, Barcelona, Spain; University of Manchester, United Kingdom

## Abstract

Representations in the cortex are often distributed with graded firing rates in the neuronal populations. The firing rate probability distribution of each neuron to a set of stimuli is often exponential or gamma. In processes in the brain, such as decision-making, that are influenced by the noise produced by the close to random spike timings of each neuron for a given mean rate, the noise with this graded type of representation may be larger than with the binary firing rate distribution that is usually investigated. In integrate-and-fire simulations of an attractor decision-making network, we show that the noise is indeed greater for a given sparseness of the representation for graded, exponential, than for binary firing rate distributions. The greater noise was measured by faster escaping times from the spontaneous firing rate state when the decision cues are applied, and this corresponds to faster decision or reaction times. The greater noise was also evident as less stability of the spontaneous firing state before the decision cues are applied. The implication is that spiking-related noise will continue to be a factor that influences processes such as decision-making, signal detection, short-term memory, and memory recall even with the quite large networks found in the cerebral cortex. In these networks there are several thousand recurrent collateral synapses onto each neuron. The greater noise with graded firing rate distributions has the advantage that it can increase the speed of operation of cortical circuitry.

## Introduction

**Figure 1:**
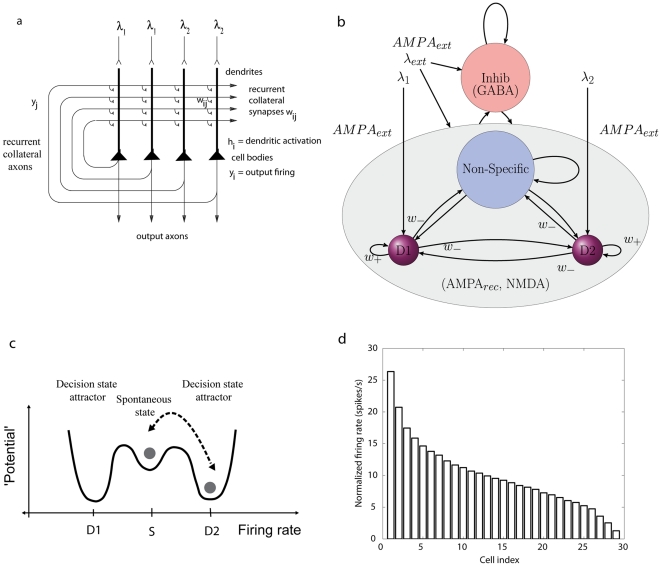
Attractor or autoassociation single network architecture for decision-making. (a) The network. The evidence for decision 1 is applied via the 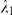 inputs, and for decision 2 via the 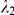 inputs. The synaptic weights 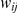 have been associatively modified during training in the presence of 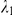 and at a different time of 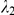. When 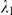 and 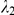 are applied, each attractor competes through the inhibitory interneurons (not shown), until one wins the competition, and the network falls into one of the high firing rate attractors that represents the decision. The noise in the network caused by the random spiking times of the neurons (for a given mean rate) means that on some trials, for given inputs, the neurons in the decision 1 (D1) attractor are more likely to win, and on other trials the neurons in the decision 2 (D2) attractor are more likely to win. This makes the decision-making probabilistic, for, as shown in (c), the noise influences when the system will jump out of the spontaneous firing stable (low energy) state S, and whether it jumps into the high firing state for decision 1 (D1) or decision 2 (D2). (b) The architecture of the integrate-and-fire network used to model decision-making (see text). (c) A multistable ‘effective energy landscape’ for decision-making with stable states shown as low ‘potential’ basins. Even when the inputs are being applied to the network, the spontaneous firing rate state is stable, and noise provokes transitions from the low firing rate spontaneous state S into the high firing rate decision attractor state D1 or D2. If the noise is greater, the escaping time to a decision state, and thus the decision of reaction time, will be shorter (see Rolls and Deco 2010). (d) The firing rates of a population of inferior temporal cortex neurons to any one stimulus from a set of 20 face and non-face stimuli. The rates of each neuron were normalized to the same average value of 10 spikes/s, then for each stimulus, the cell firing rates were placed in rank order, and then the mean firing rates of the first ranked cell, second ranked cell, etc. were taken. The graph thus shows, for any one stimulus picked at random, the expected normalized firing rates of the population of neurons. (Panel (d) after Franco, Rolls, Aggelopoulos and Jerez 2007.).

If an autoassociation or attractor network is provided with two or more inputs, as illustrated in Fig. 1a and b, each biasing an attractor population of neurons with large intra-population excitatory connection strengths, then this forms a biased competition model of decision-making in which a high firing rate of one of the possible attractor states represents a decision [1]–[4]. An attractor state is a stable high firing rate state of one of the populations of neurons, and nearby firing rate patterns in the space are attracted towards the firing rates specified by the connection strengths between the neurons in the winning population [4]–[6].

Many processes in the brain are influenced by the noise or variability of neuronal spike firing [4], [7], [8]. The action potentials are generated in a way that frequently approximates a Poisson process, in which the spikes for a given mean firing rate occur at times that are essentially random (apart from a small effect of the refractory period), with a coefficient of variation of the interspike interval distribution (CV) near 1.0 [4], [9]. The sources of the noise include quantal transmitter release, and noise in ion channel openings [7]. The membrane potential is often held close to the firing threshold, and then small changes in the inputs and the noise in the neuronal operations cause spikes to be emitted at almost random times for a given mean firing rate. Spiking neuronal networks with balanced inhibition and excitation currents and associatively modified recurrent synaptic connections can be shown to possess a stable attractor state where neuron spiking is approximately Poisson too [10], [11]. The noise caused by the variability of individual neuron spiking which then affects other neurons in the network can play an important role in the function of such recurrent attractor networks, by causing for example an otherwise stable network to jump into a decision state [2], [4]. The noise in the operation of the system makes the decision-making process non-deterministic, with the system choosing one of the attractor states with a probability that depends on the relative strengths of the different input biases 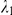, 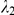 etc [1], [2]. The randomness or stochasticity in the operation of the system can be advantageous, not only by providing a basis for probabilistic decision-making in which each decision will be sampled in a way that depends on the relative strengths of the inputs, but also in memory recall which by being probabilistic allows different memories to be recalled from occasion to occasion, helping with creative thought processes as these become non-deterministic, and with signal detection which can become more sensitive than a fixed threshold system in the process known as stochastic resonance [4].

For these advantageous stochastic processes to be realized in the brain, the amount of noise must be significant. One factor that affects the amount of noise is the number of neurons in the fully connected network. As the number of neurons approaches infinity and if their responses are uncorrelated, the noise or statistical fluctuations caused by the neuronal firing decreases to zero, and the mathematically convenient mean-field approximation holds, allowing many properties of the system to be calculated analytically [1], [2], [4], [12]. Using integrate-and-fire attractor network simulations of decision-making which include the spiking-related noise, we have shown that the stochastic fluctuations in a finite-sized system are still a significant influence to produce probabilistic decision-making with networks with 4096 neurons and 4096 synapses per neuron [2]. This is biologically relevant in that neocortical neurons are likely to have in this order (4,000–9,000) of recurrent collateral excitatory connections from other pyramidal cells [13]–[16].

Another factor that may influence the noise is the distribution of the firing rates of the population of neurons. In most analyses of integrate-and-fire attractor neuronal networks, a binary distribution of the firing rates of the neuronal populations is used, partly because this is consistent with the mean-field approximation that allows analytic calculation [1], [2], [4], [12], [17], and partly because the code is simpler and more efficient. With a binary firing rate distribution, a proportion of the neurons has the same high rate, and the remainder have a low rate. The sparseness of the representation can then be defined as the proportion of neurons with a high rate, that is, the proportion of the neurons in any one of the attractors stored in the network [16], [18], [19]. However, representations in the brain are not binary, with one or a number of neurons with the same high firing rate for any one stimulus, and the remainder of the neurons with a low spontaneous rate of firing. Instead representations provided by populations of neurons in the brain are often graded with firing rates in which for each stimulus or event a few neurons fire fast, and more and more neurons fire with lower rates [16]. This has been found for representations of visual stimuli in the inferior temporal visual cortex [20]–[22] and the primary visual cortex [22]; of olfactory stimuli in the orbitofrontal cortex [23]; of taste and oral texture stimuli in the primary taste cortex [24], orbitofrontal cortex [25], [26] and amygdala [27], [28]; and of spatial view in the primate hippocampus [29]. The firing rate probability distribution of each neuron to a set of stimuli is often exponential (or gamma if there is higher spontaneous activity) [22], [30], [31]. Across a population of neurons, the probability distribution of the firing rates for any one stimulus is also close to exponential [30], [31]. The graded nature of the firing rates of a population of inferior temporal neurons to one stimulus from a set of 20 stimuli is illustrated in Fig. 1d [16], [31].

The important question that then arises is how the noise present in a graded population firing rate representation, as frequently found in the brain, compares with the binary firing rate representations. In this paper we investigate this by developing new integrate-and-fire simulations of neuronal networks that allow graded, close to exponential as found in the brain, representations to be used, and then measuring the time taken to reach a decision, which measures the noise-influenced escaping time from the spontaneous state, as illustrated in Fig. 1c [4]. We perform this investigation in a system in which the spontaneous state, even when the decision cues are being applied, is stable, so that it is only noise that provokes an escape from the spontaneous state to a high firing rate attractor state. We are careful to control the sparseness of the graded rate representation, to allow direct comparison with the binary representation. We show that there is more noise with graded as compared with binary rate representations. We draw out the implications for understanding noise, decision-making, and related phenomena in the brain. The implications include the fact that, given that graded rate representations are more noisy than binary rate representations, spiking-related stochastic dynamics will continue to be a principle of brain function that makes a contribution even up to realistically large neuronal networks as found in the brain, with in the order of thousands of recurrent collateral synapses onto each neuron [4].

The amount of noise in neuronal networks that are biologically realistic and its effects on the stability of the networks is an important issue with medical, societal, and economic impact, for recent approaches to schizophrenia and obsessive-compulsive disorder have suggested that a contribution to these states is too little and too much stability respectively [32]–[36]. The research described here is very relevant to this issue, for it investigates how much spiking-related noise there is with graded firing rate distribution representations (which are found in the brain [16], [20], [30], [31], [37]), rather than the binary firing rate distribution systems more commonly studied [1], [2], [4], [38], [39].

## Methods

### The integrate-and-fire attractor neuronal network model of decision-making

The probabilistic decision-making network we use is a spiking neuronal network model with a mean-field equivalent [1], but instead set to operate with parameters determined by the mean-field analysis that ensure that the spontaneous firing rate state is stable even when the decision-cues are applied, so that it is only the noise that provokes a transition to a high firing rate attractor state, allowing the effects of the noise to be clearly measured [2], [4]. The reasons for using this particular integrate-and-fire spiking attractor network model are that this is an established model with (in the binary case) a mean-field equivalent allowing mathematical analysis; that many studies of short-term memory, decision-making and attention have been performed with this model which captures many aspects of experimental data (in a number of cases because, for example, NMDA receptors are included); and that it captures many aspects of cortical dynamics well [1]–[4], [12], [32], [34], [38]–[42].

The fully connected network consists of separate populations of excitatory and inhibitory neurons as shown in Fig. 1. Two sub-populations of the excitatory neurons are referred to as decision pools, ‘D1’ and ‘D2’. The decision pools each encode a decision to one of the stimuli, and receive as decision-related inputs 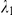 and 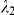. The remaining excitatory neurons are called the ‘non-Specific’ neurons, and do not respond to the decision-making stimuli used, but do allow a given sparseness of the representation of the decision-attractors to be achieved. (These neurons might in the brain respond to different stimuli, decisions, or memories.) A description of the network follows, and we further provide a description according to the recommendations of [43] in the Supplementary Material S1.

In our initial simulations, the network contained 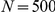 neurons, with 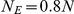 excitatory neurons, and 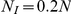 inhibitory neurons. The two decision pools are equal size sub-populations with the proportion of the excitatory neurons in a decision pool, or the sparseness of the representation with binary encoding, 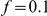, resulting in the number of neurons in a decision pool 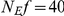. The neuron pools are non-overlapping, meaning that the neurons in each pool belong to one pool only.

We structure the network by establishing the strength of interactions between pools to take values that could occur through a process of associative long-term potentiation (LTP) and long-term depression (LTD). Neurons that respond to the same stimulus, or in other words ones that are in the same decision pool, will have stronger connections. The connection strength between neurons will be weaker if they respond to different stimuli. The synaptic weights are set effectively by the presynaptic and post-synaptic firing rate reflecting associative connectivity [16]. In the binary representation case neurons in the same decision pool are connected to each other with a strong average weight 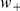, and are connected to neurons in the other excitatory pools with a weak average weight 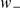. All other synaptic weights are set to unity. Using a mean-field analysis which applies to the binary firing rate distribution case [2], we chose 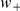 to be near 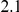, and 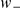 to be near 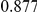 to achieve a stable spontaneous state (in the absence of noise) even when the decision cues were being applied, and stable high firing rate decision states. In particular, 
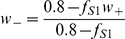
 [1], [2], [4], [12], [32].

### Neuron model

Neurons in our network use Integrate-and-Fire (IF) dynamics [1], [2], [4], [12], [44], [45] to describe the membrane potential of neurons. We chose biologically realistic constants to obtain firing rates that are comparable to experimental measurements of actual neural activity. IF neurons integrate synaptic current into a membrane potential, and then fire when the membrane potential reaches a voltage threshold. The equation that governs the membrane potential of a neuron 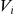 is given by
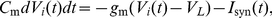
(1)where 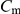 is the membrane capacitance, 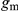 is the leak conductance, 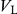 is the leak reversal potential, and 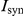 is the total synaptic input. A spike is produced by a neuron when its membrane potential exceeds a threshold 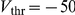 mV and its membrane potential is reset to a value 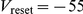 mV. Neurons are held at 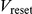 for a refractory period 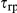 immediately following a spike.

### Synapses

The synaptic current flowing into each neuron is described in terms of neurotransmitter components. The four families of receptors used are GABA, NMDA, AMPA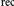, and AMPA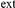. The neurotransmitters released from a presynaptic excitatory neuron act through AMPA and NMDA receptors, while inhibitory neurons activate ion channels through GABA receptors. Each neuron in the network has 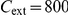 external synapses that deliver input information and background spontaneous firing from other parts of the brain. Each neuron receives via each of these 800 synapses external inputs a spike train modeled by a Poisson process with rate 3.0 Hz, making the total external input 2400 Hz per neuron.

The synaptic current is given by a sum of glutamatergic, AMPA (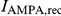) and NMDA (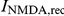) mediated, currents from the excitatory recurrent collateral connections; an AMPA (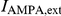) mediated external excitatory current; and an inhibitory GABAergic current (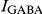):
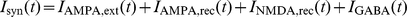
in which
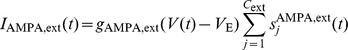


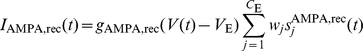


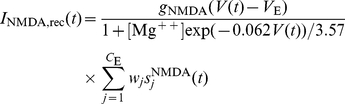


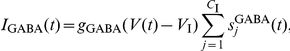
where 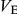 and 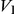 are reversal potentials for excitatory and inhibitory PSPs, the 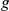 terms represent synaptic conductances, 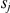 are the fractions of open synaptically activated ion channels at synapse 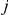, and weights 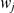 represent the structure of the synaptic connections. (The index 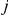 above refers to different synapses, external, recurrent, AMPA, NMDA, GABA etc as indicated.)

Post-synaptic potentials are generated by the opening of channels triggered by the action potential of the presynaptic neuron. As mentioned above, the dynamics of these channels are described by the gating variables 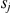. The dynamics of these variables are given by
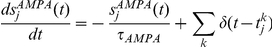


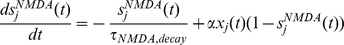


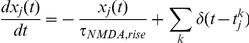


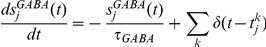
where the sums over 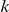 represent a sum over spikes formulated as 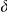-Peaks (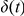) emitted by presynaptic neuron 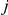 at time 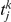.

The constants used in the simulations are shown in Table 1.

**Table 1:** Simulation constants.

**Global constants**			
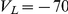 mV	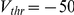 mV	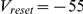 mV	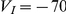 mV
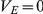 mV	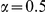 ms 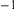	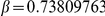	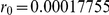
**Inhibitory neuron constants**			
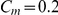 nF	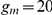 nS	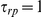 ms	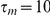 ms
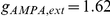 nS	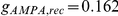 nS	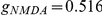 nS	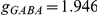 nS
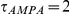 ms	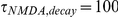 ms	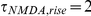 ms	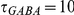 ms
**Excitatory neuron constants**			
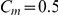 nF	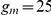 nS	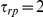 ms	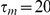 ms
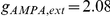 nS	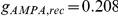 nS	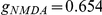 nS	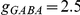 nS
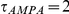 ms	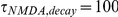 ms	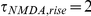 ms	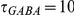 ms

### Graded Weight Patterns

In an attractor network, the synaptic weights of the recurrent connections are set by an associative (or Hebbian) synaptic modification rule with the form
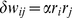
(2)where 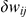 is the change of synaptic weight from presynaptic neuron 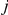 onto postsynaptic neuron 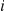, 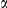 is a learning rate constant, 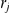 is the presynaptic firing rate, and 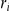 is the postsynaptic firing rate when a pattern is being trained [5], [16], [46]. To achieve this for the firing rate distributions investigated, we imposed binary and graded firing rates on the network by selecting the distribution of the recurrent synaptic weights in each of the two decision pools. To achieve a binary firing pattern all the weights within a decision pool were set uniformly to the same value 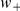.

Graded firing patterns were achieved by setting the synaptic weights of the recurrent connections within each of the decision pools to be in the form of a discrete exponential-like firing rate (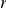) distribution generated using methods taken from [47].
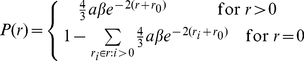
(3)where 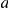 is the sparseness of the pattern defined in Equation 4, and 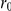 is the firing rate of the lowest discretized level. In simulations we use 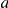 = 0.1 to correspond to the fraction of excitatory neurons that are in a single decision pool. We chose 10 equal-spaced discretized levels to evaluate the distribution (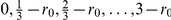). 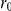 and 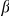 are chosen so that first and second moments of the firing rate distribution are equal to the sparseness, i.e. 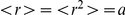, see Table 1. A weight matrix 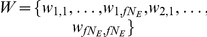 was constructed by first sampling a firing rate for each neuron, 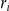, using Equation 3 and then setting 
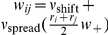
. 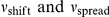 are two free parameters used to fine control the firing activity of the network.

### Sparseness

The population sparseness 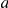 of a binary representation is the proportion of neurons active to represent any one stimulus or decision in the set. The sparseness can be generalized to graded firing representations as shown in Equation 4
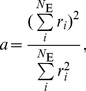
(4)where 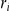 is the firing rate measured for neuron 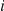 in the population of 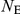 excitatory neurons in the network [16], [18], [31], [48]. We note that this is the sparseness of the representation measured for any one stimulus over the population of neurons [16], [31]. For the sparseness values shown in this paper, the average firing rate of a neuron across all trials was calculated, and then the population sparseness of this set of firing rates was measured.

### Simulation regime

The network was simulated numerically using a second order Runge-Kutta algorithm step with an integration step 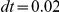 ms for a time period of 4 seconds. First there was a 2 s baseline period of spontaneous activity in which 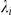 = 3.0 Hz for each of the 800 external synapses onto each neuron. There was then a 2 s decision period in which the decision stimuli were applied by increasing the firing rates for the 800 external input synapses on each of the neurons in the two decision pools to 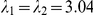 Hz (an extra 32 Hz per neuron). During the decision period, the noise in the network, and the increased firing rate bias functioning as a decision cue to each decision pool of neurons, causes one of the decision populations of neurons to jump to a high firing rate attractor state with the assistance of the positive feedback in the recurrent collaterals. This high firing inhibits through the inhibitory interneurons the other decision population of neurons. There is thus a single winning population on each trial, and which of the two populations wins on a particular trial is determined by the statistical fluctuations in the firing rates of the neurons in each decision population, and the difference in the two inputs 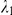 and 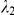, i.e. 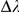.

## Results

**Figure 2:**
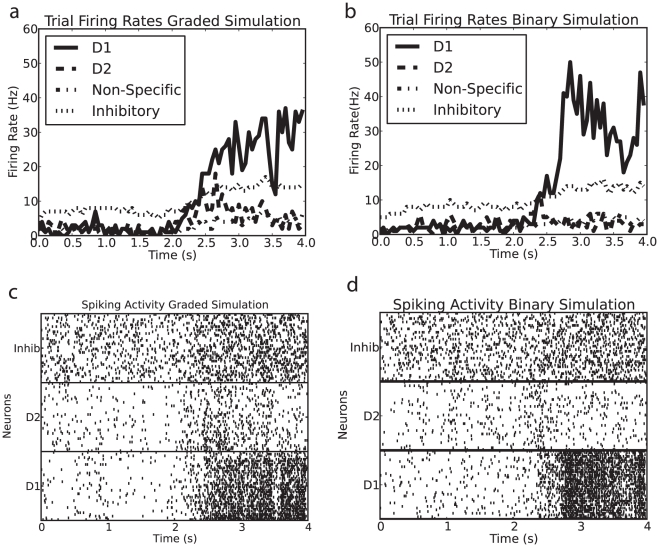
Examples of single trials of the simulations for graded and binary firing rate representations. a,b: Example of the average firing rates for the different pools on a single trial for (a) the graded firing rate simulations and (b) the binary firing rate simulations. (c) and (d): the rastergrams for the corresponding trial, with each row of the rastergram providing the spike times for one of 40 neurons in each pool. In the case of the graded simulation, the neurons with the higher firing rates are plotted in the lower rows for each population of neurons. There is a 2 s period of spontaneous activity from 0–2 s, and then the decision cues are applied to the neurons in pools D1 and D2 from time = 2–4 s.

The operation of the system is illustrated for a single trial in Fig. 2 which shows that for both the binary case and the graded firing rate distribution case the neurons in the winning pool have an average firing rate greater than 25 Hz.

### Firing rate distribution

**Figure 3:**
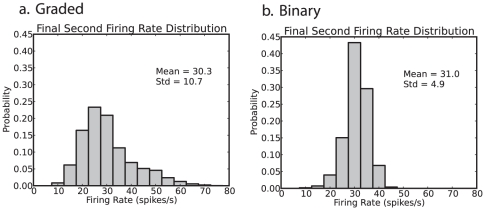
Firing rate probability distributions of the winning pool measured from the final 1 s of the simulations for (a) graded and (b) binary firing rate distributions.

Fig. 3a,b shows for the graded (a) and binary (b) rate distribution simulations the firing rate the firing rate probability distributions achieved by the weight matrix we selected. The firing rates were measured in the last 1 s of the simulation (time = 3–4 s). The distribution of firing rates for the binary case has low variance, with nearly identical mean firing rates for each of the individual neurons in the winning pool. In contrast, the graded rate distribution simulations show more variation in the distribution. The exponential-like shape occurs in both the spontaneous and decision states, but is more pronounced in the decision state. The parameters were set to achieve this set of graded distribution firing rates, rather than a perfectly exponential distribution, because we wished to ensure that the mean firing rate and sparseness of the representation were similar in the binary and graded rate distribution cases, while at the same time having clearly graded distribution firing rates for the graded simulations so that the effects of graded vs binary firing rate distributions could be measured under conditions where the mean rate, and the sparseness, were essentially identical. The mean firing rates for the graded case (a) were 30.3 spikes/s and for the binary rate distribution case were 31.0 spikes/s, showing that the parameters for the recurrent weights had been selected to make the firing rates very similar in these two cases. This was an aim, as higher firing rates can reflect increased excitation in the network which could decrease decision times. As was also an aim, the standard deviation of the firing rate distribution was higher for the graded case (10.7 Hz) than for the binary case (4.9 Hz).

As the sparseness of the representation might influence the noise in the network and the measured decision time (with sparse representations with small values of 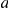 expected to be more noisy), we were careful to ensure that the sparseness of the representation for the binary and graded cases were similar. (They were set by the choice of the recurrent synaptic weights in the two decision populations, which is the distribution that produced the graded firing rates.) The sparseness measured using Equation 4 from both sets of simulations was very similar. The final steady state value with one of the pools in its winning attractor state was close to the theoretical value of 0.1, due to there being 40 neurons in each decision pool in a population of 400 excitatory neurons.

The variability of the firing was measured by computing the coefficient of variation (CV) of the firing rates for single neurons (using 50 ms bins) for different temporal periods. The CV measured in the second before the decision cues were applied (the period of spontaneous firing) was 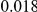 for the binary and was 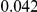 for the graded rate distributions. In the final second of simulation for the winning attractor the CV was 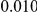 for the binary and was 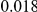 for the graded rate distributions. The variability by this measure was consistently higher for the graded simulations than for binary rate distributions.

### Decision time

An important measure of the noise in the system is the escaping time of the system after the decision cues are applied from the spontaneous state to a decision state. Increased noise will decrease the escaping time, and thus the decision or reaction time, as illustrated in Fig. 1c. 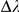 was 0 Hz per neuron for these simulations.

**Figure 4:**
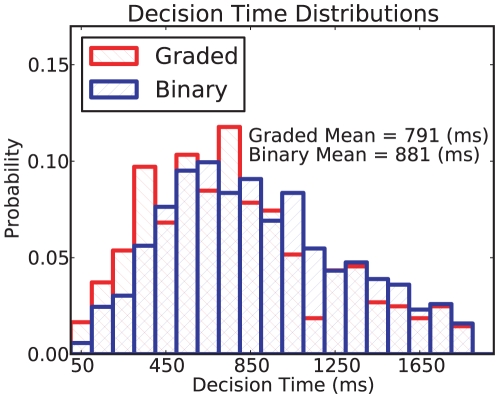
Decision times. Histograms of decision times for 1000 graded and 1000 binary firing rate distribution simulations. The criterion for a decision time was that the average firing rate of one decision pool should be 25 Hz higher than that of the other decision pool for three consecutive 50 ms periods. 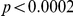 for graded vs binary firing rate distributions using Kolmogorov-Smirnov tests, t-tests, and Mann-Whitney U tests of the two distributions. The mean decision time was 791
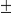
430 (sd) ms for the graded case, and 881
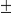
420 ms for the binary case.

To address the issue of the amount of noise in the system with graded vs binary firing rate distribution representations, we show in Fig. 4 the decision times of the network with graded and binary rate distribution representations. The decision (or reaction) time was measured by the time it took from the time at which the decision cues were applied (t = 2 s) when the network was in the spontaneous firing rate baseline state for one of the decision pools to fire 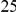 Hz higher than the other one for 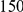 ms. The important result is that the graded firing rate distribution patterns produce significantly (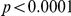) faster reaction times (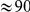 ms), than the binary firing rate distribution patterns (Fig. 4). (The non-parametric Mann-Whitney U and Kolmogorov-Smirnov tests were used in all cases to test for differences in the decision time distributions.) The mean decision time was 881 ms for the binary firing rate representations, and 791 ms for the graded rate distribution representations. Further analysis showed that the number of trials required for these decision times to become significantly shorter (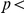
0.05) for the graded compared to the binary rate distribution representation was on average 541 trials.

**Figure 5:**
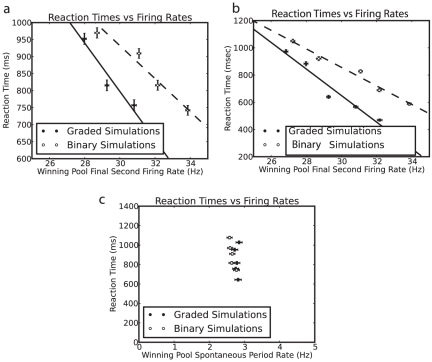
Decision times. (a) Decision times of 1000 simulations for each point with a shifted 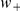 parameter and thus different firing rates for the winning pool in the final second, for the graded and binary firing rate distribution cases. b. The same as (a) except that distributions corrected for premature decisions were used (see text). The error bars signify the estimated standard error of the firing rate and reaction time. (c) The same plot as (a) except that the firing rate is measured during the spontaneous period.

The faster decision times for the graded firing rate distributions (Fig. 4) were found when the mean firing rates in the attractor state, and the sparseness of the representation, were carefully matched in the graded and binary rate distribution simulations. We further showed that it was not a faster firing rate for the graded rate distribution simulations that accounted for the faster decision times for the graded firing rate distribution by performing a whole series of further simulations (each with 1000 trials) in which the parameters of the recurrent synaptic weights between the neurons in a decision pool were systematically varied to obtain decision times for the graded and binary firing rate distribution cases that bracketed each other. It is clear (Fig. 5a) that while increases of 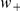 that increased the firing rates when in the winning attractor did decrease the mean decision time of the decision-making process, for any given mean firing rate of the neurons in the winning attractor, the decision times were faster for the graded than for the binary firing rate distributions. The faster decision times for the graded than for the binary firing rate distributions are statistically significant and robust across different firing rates of the winning pool (Fig. 5a).

**Figure 6:**
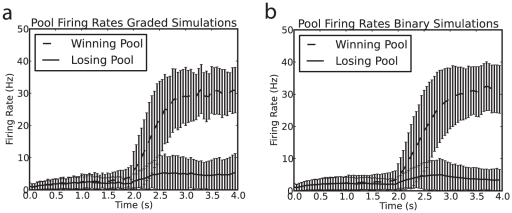
Similar firing rates for (a) graded and (b) binary simulations. Mean firing rates over 1000 trials for the winning and losing pools in graded (a) and binary (b) firing rate distributions. The decision cues were turned on at t = 2 s. The error bars show the standard deviations. The winning pool is chosen to be the pool with average firing rate 10 Hz greater than the competing pool in the last 1 s of the simulation. 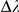 was 0 Hz for these simulations.

Further evidence on this follows. The graded firing rate distribution simulations tended to have a higher firing rate for the winning pool when simulations were run across distributions with the same average synaptic weight between the neurons in a decision pool. We chose 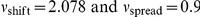 to find a winning firing rate and sparseness that were close for both distributions for the results illustrated in Fig. 4. The average firing rates for these values of the parameters are shown in Fig. 6. The similar firing rates for the winning pools during the spontaneous baseline and decision periods are shown.

As analyzed in Section simulations with graded compared to binary firing rate distributions showed an alteration in their stability when in the spontaneous firing rate state before the decision cues were applied. A contribution to the decreased decision times could be that the graded rate distribution simulations destabilized not due to the applied cues, but rather became unstable in the baseline spontaneous firing rate in the period before the decision cues were applied. For example, in 1000 trials we ran with a network size 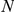 = 500, on 149 trials the firing jumped into or towards a decision state early, by 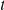 = 2 s, in the binary case. This has been described previously for similar parameters of the system [32], [33], [49]. We excluded from the decision time analysis those trials that transited into or towards a decision state before the decision cues were applied at 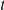 = 2 s. The criterion was that trials were excluded if the mean rate of a decision pool exceeded 10 spikes/s in the half second before the decision cues were applied. What we did find in the present simulations was that with the graded firing rate distribution simulations, there were more trials, 270, in which the spontaneous state was unstable, in that there was a noise-provoked transition into a decision state before the decision cues were applied at 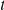 = 2 s. To correct for this possible effect we subtracted a reaction time distribution without the application of decision cues from the distribution with decision cues. Simulations were repeated with the same parameters, except that no cues were applied. The distribution of the reaction times of these ‘no cues’ simulations was computed. The ‘corrected distributions’ were computed by subtracting the number of times the ‘no cues’ simulation reacted in a given period from the number of times the simulation reacted in the same period in the ‘with cues’ simulations. This provided a decision time distribution that is corrected for the possibility of simulation trials jumping purely from the baseline spontaneous rate to a high firing rate state. When this correction is applied, we still observed that the decision times are faster for the graded than for the binary firing rate distribution cases, as shown in Figs. 4c,d and 5b.

In summary, faster decision times are found with graded than with binary firing rate distributions, and this is not likely to be due to any increase in firing rate during the spontaneous period, nor is it due to faster firing rates during the decision-making period.

**Figure 7:**
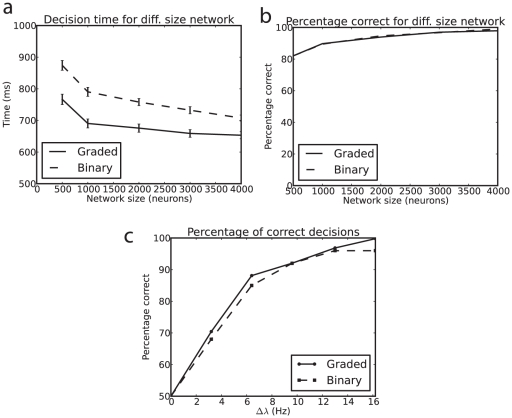
Decision times for networks of different sizes with graded and binary firing rate representations. (a) Decision times of 500 simulations for networks of different size 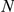, the number of neurons in the network, for the graded and binary firing rate distribution cases. The means and standard deviations are shown. 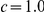 (fully connected). Gradation level 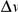 = 0.81. 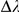 = 16 Hz. (b). The percentage correct for networks of different size 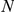, the number of neurons in the network, for the graded and binary firing rate distribution cases. 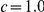. Gradation level 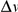 = 0.81. 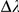 = 16 Hz. (c) The percentage correct for 500 simulations with different values of 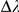; 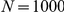, 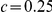, and Gradation level 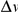 = 2.1.

**Figure 8:**
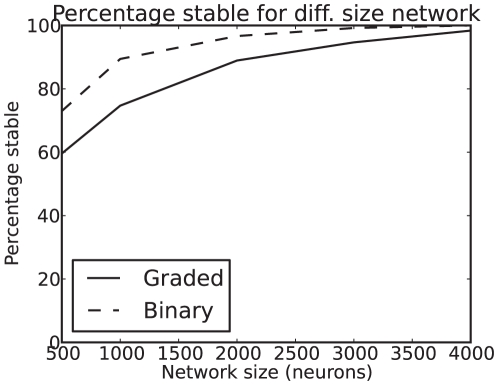
Less stability for graded than for binary firing rate distributions. The percentage of trials in which the spontaneous state was stable for the graded and the binary firing rate distribution cases for networks of different size 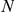, the total number of neurons in the network. The lower curve is for the graded case.

So far, the results presented have been for a network of size 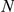 = 500 neurons in the network. To investigate whether the decision times remain shorter for the graded than the binary firing rate distributions as the network becomes larger, an important issue as networks in the cerebral cortex typically have in the order of thousands of recurrent collateral synaptic connections onto each neuron [16], we performed further simulations with larger 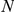. Fig. 7a shows that for each size of network up to 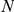 = 4000, the decision time is shorter for the graded than for the binary firing rate distribution cases. The performance in terms of the percentage correct was similar for the graded and binary rate distribution cases for different network sizes, as shown in Fig. 7b, so there is no penalty in terms of decision accuracy of the faster decision times found with networks with graded than with binary firing rate distributions. An important aspect of this result is that the larger networks are quite stable in the spontaneous period (as shown in Fig. 8), and this is further evidence that instability of the spontaneous state is not crucial to the faster decision times of the networks with graded than with binary firing rate distributions. (For example, with 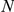 = 4000, 98% of the trials in the graded rate distribution case were stable in the spontaneous period (and were excluded from the analysis), and we still found faster decision times when the decision cues were applied for the graded firing rate distributions, as shown in Fig. 7a.).

### Stability of the spontaneous state

Noise and the positive feedback in this system can cause the network to jump into a decision state from the spontaneous state even before the decision cues are applied (at 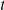 = 2 s in our simulations). We analyzed the stability for the graded vs binary firing rate distribution cases by measuring the percentage of trials on which the binary and graded firing rate distribution simulations transited into or towards a high firing rate decision state before the decision cues were applied at 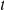 = 2 s. The parameters for the binary simulation had been set with the mean field analysis so that the mean spontaneous firing rate should be 3 spikes/s. The criterion for instability of the spontaneous state was that the mean rate of either decision pool exceeded 5 spikes/s in the 250 ms before the decision cues were applied. Fig. 8 shows the percentage of trials on which the spontaneous state was stable for the graded and the binary firing rate distribution cases for networks of different size 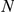, the total number of neurons in the network. As expected, the larger in terms of 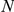 the network becomes, the more stable the network becomes, as the finite size of the network becomes less of a factor. (In the mean field case, or with an infinite number of neurons in the spiking simulations, the noise effects would diminish to zero.) Fig. 8 shows that the network with the graded firing rate distribution is for each value of 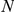 less stable in the spontaneous period than the network with the binary firing rate distribution.

This effect was not accounted for by any increase in the mean spontaneous firing rates of the decision pool neurons in the graded firing rate distribution case, which remained at a mean value of approximately 3 Hz as shown in Fig. 6 (unless a noise-provoked transition occurred) because 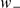 was decreased to compensate for any increase in 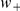 by using the procedure described previously [1], [2], [4], [12], [32]. Indeed, the results in Fig. 5c show that the firing rate during the spontaneous period does not respond to changes in the 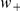 parameter because it is compensated for by changes in the 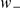 parameter. These results are consistent with the mean-field theory developed by [12], who set up a system in which changes in 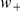 will only change the firing rates during the decision state, not during the spontaneous state. Moreover, the sparseness of the representation was the same for the graded and binary firing rate distribution cases.

The results on stability during the spontaneous state thus provide further evidence that the network with graded firing rate distributions is more noisy than the network with binary firing rate distributions for the decision pools, even when the mean rates and sparsenesses are the same.

### Noise in the system: the variance of the firing rates of the neurons

**Figure 9:**
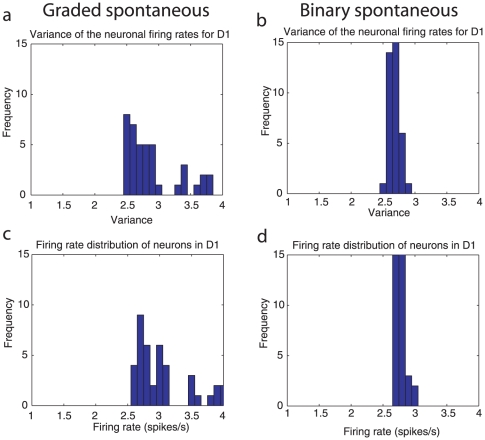
The firing rate and variance distributions for the graded (left) and binary (right) firing rate distributions in the spontaneous period before the decision cues were applied. The distribution for the set of 40 neurons in decision pool 1 of the variance across trials of the firing rate in the spontaneous period (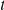 = 1.5–2.0 s) for a network of size 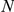 = 500 for the graded (a) and binary (b) cases. The variance for each neuron across trials is that of the firing rates measured for a 50 ms bin during the spontaneous period with 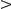
550 trials with stable spontaneous firing rates using the criterion described in the text. The average variance for each neuron over 10 bins from 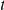 = 1.5–2.0 s is shown. (c, d) Firing rate probability distributions for the spontaneous firing rate in the same period for the graded (c) and binary (d) cases.

Another measure of the noise in the system is the variance of the firing rates of the neurons in a decision pool during decision-making. If some of the neurons in a pool have more variance, that pool may be more likely to cross a bifurcation from the spontaneous firing rate state and to enter a decision state without any decision cue, or to make a decision after the decision cues have been applied more rapidly (cf. Fig. 1). Fig. 9 shows the distribution for the 40 neurons in decision pool 1 of the variance across trials of the firing rate in the spontaneous period (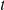 = 1.5–2 s) for a network of size 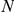 = 500 for the graded (a) and binary (b) rate distribution cases. The variance is that for each neuron across trials of the firing rates measured in a 50 ms bin during the spontaneous period with 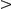
550 trials with stable spontaneous firing rates using the criterion described above. The average variance for each neuron over 10 bins from 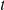 = 1.5–2.0 s is indicated. The variance distribution reaches higher values for some neurons with the graded than with the binary distribution, and this is just consistent with the approximately Poisson firing of the neurons (with which the variance = the mean), and the fact that the firing rate distribution shows some neurons with relatively high firing rates (up to 4 spikes/s) with the graded representation in the pre-cue period, as shown in Fig. 9c and d. We emphasize that the mean firing rates and variances are very similar for the binary and graded rate distribution cases: it is the distributions that are different, as shown in Fig. 9. The concept here is that for the graded rate distribution representation the subset of neurons with higher than average variance (and firing rates) contribute especially strongly to the noise (i.e. variation, fluctuation) in the system that promotes diffusion [50] across the barrier in the energy landscape (Fig. 1), and that the effect of these neurons is helped by their stronger than average connection weights to other neurons within their decision pool, which enable statistical fluctuations in their rates to be felt especially strongly by the other neurons in the same decision attractor.

### Performance with graded firing rate distributions and diluted connectivity

Up to this point, the network was studied with fully connectivity of its neurons. In order to investigate a more biologically plausible scenario, we conducted simulations with diluted connectivity. In order to keep the mean input to each neuron the same in diluted simulations as it was in fully connected simulations, for the diluted connectivity we kept the same number of connections 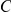 per neuron as in the fully connected network, but increased the number of neurons in the decision pools. We parameterized dilution by a connectivity level, 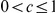. 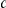 = 1 corresponds to the fully connected case. Diluted networks with dilution 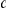 would have the number of neurons in the decision pool set to 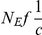. The 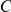 connections to a neuron were received from a randomly selected set of the 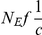 neurons in the same decision pool.

We measured decision times for two values of 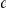. As described elsewhere [51], smaller values of 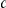 resulted in slower decision times. One of the new findings reported here is that for diluted connectivity, graded firing rate representations produced faster decision times than binary rate distribution representations. In particular, for 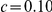, the mean decision time in the graded case was 1124 ms (SE 33 sd 335 ms), and in the binary case it was 1192 ms (SE 32 sd 320 ms). For 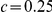, the mean decision time in the graded case was 984 ms (SE 16 sd 345 ms), and in the binary case it was 1077 ms (SE 15 sd 332 ms) (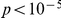).

In summary, in networks with diluted connectivity, just as in fully connected networks, graded firing rate distribution representations produced faster decision times than binary firing rate representations. This is consistent with more noise in attractor networks with graded than with binary firing rate distribution representations.

### Performance during decision-making with 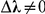 Hz

So far we have shown results mainly for 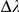 = 0 Hz, that is when the inputs during the decision-making period to D1 and D2 are equal. The performance of the network is close to the expected 50% correct, that is D1 wins on approximately 50% of the trials, and D2 on approximately 50% of the trials. However, the evidence for the two decisions is often not equal, and in this section we consider whether when running with 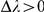, different effects occur. For example, if the graded rate distribution system is more excitable and responds faster than the binary rate distribution system, there might be a speed-accuracy tradeoff of the type investigated for many decades in psychology [52]. It would be of interest if for example the graded rate distribution system with its faster decision times was less accurate (in terms of percentage correct), though also interesting if it maintained its accuracy even when the reaction times were faster.

Fig. 7a shows that for different sizes of network up to 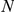 = 4000, the decision time is shorter for the graded than for the binary firing rate distribution cases with 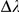 = 16 Hz per neuron. The performance in terms of the percentage correct was similar for the graded and binary rate distribution cases for different network sizes, as shown in Fig. 7b, and for different values of 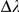 as shown in Fig. 7c, so there is no penalty in terms of decision accuracy of the faster decision times found with networks with graded than with binary firing rate distributions within these parameter ranges.

### Performance for different levels of firing rate gradation

Up to this point we have only presented graded rate distribution simulations with a value of gradation that was small enough to keep the firing rates and stability close to the results in the binary simulations. We have in addition simulated networks with higher amounts of gradation. We parameterized the amount of gradation in the network by 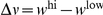, where 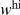 is the highest recurrent weight, and 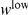 was the lowest recurrent weight. The other results in this paper have 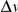 approximately 0.81. In further investigations With moderate dilution, 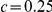, and 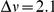, decision times decreased to a mean value of 445 ms, and stability during the spontaneous period was reduced to 37%, compared to a mean decision time of 984 ms (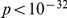) and 98% stability for the same simulation but with 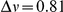.

Thus increasing the range of firing rates in the graded distribution representation decreased the decision time and decreased the stability of the spontaneous firing rate state. This is evidence that increasing the range of the firing rate distributions introduces more noise into the neuronal network.

### Noise with graded firing rate distribution representations in larger networks

As spiking attractor networks are increased in size, the statistical fluctuations caused by the close to Poisson spiking times of the neurons become smaller, until with an infinite number of neurons the noise becomes 0 [4]. We have shown that in practice, measures of the noise such as the decision (escaping) time do decrease as the number of neurons is increased to 4000, but that there is still noise due to the spiking fluctuations with this size of network, in which 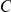 = 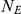 = 3200 [2], [38], [39]. However, the number of connections 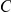 for the recurrent collateral synapses which provide for the attractor dynamics is in the order of 9,000 in the neocortex, and 12,000 in the CA3 neurons in the hippocampus [16]. To check that the findings in the present paper apply in principle to these larger networks, we were able to perform further simulations with as many as 8000 neurons in the network, which then had 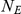 = 6400 excitatory neurons, and 6400 recurrent collateral synapses onto each excitatory neuron.

We simulated scaled up networks with 8000 neurons, and therefore 320 neurons in each specific decision population. With 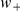 left at 2.1 as in the earlier simulations, the decision times were faster with the graded (mean 947 sd 332 ms) than with the binary (mean 1073 sd 312 ms) firing rate distributions (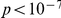 with 320 trials). Thus graded firing rate distributions do introduce more noise into the system than binary firing rate distributions, even with large networks that are the same order as the size of networks found in the cerebral cortex. Further analysis showed that these decision times became significantly shorter (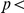
0.05) for the graded compared to the binary rate distribution representation with on average 21 trials.

With 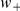 = 2.1 and 8000 neurons, the spontaneous state was much more stable, and indeed there were no unstable trials in the spontaneous period for the graded and for the binary rate distribution representations. To test whether the graded rate distribution was inherently more unstable in the spontaneous state even at this large size of network, we ran further simulations with 8000 neurons, but with 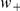 = 2.25 to promote more instability. This revealed more instability with the graded (only 87% stable) than with the binary firing rate distribution representations (97% stable, 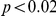).

## Discussion

In integrate-and-fire simulations of an attractor decision-making network, we have shown that the noise is greater for a graded than for a binary firing rate distribution of the populations of neurons. The noise effect was measured by faster escaping times from the spontaneous firing rate state when the decision cues are applied, and this corresponds to faster decision or reaction times (Figs. 4, 5 and 7). We note that the variability in human choice reaction times is rather large [53], [54], and this is a property that is captured by this biologically-based approach to decision-making, and memory recall [4], [39], [51].

The greater effect of the noise with the graded firing rate distributions was also measured as greater instability of the spontaneous firing rate state before any decision cues were applied (Fig. 8), that is by more noise-provoked transitions from the spontaneous state which was shown to be a stable state in the mean-field analysis in which there is no noise. The conclusion is that spiking-related noise stochastic dynamics will continue to be a principle of cortical computation that influences processes such as decision-making, signal detection, short-term memory, and memory recall even with the quite large networks found in the cerebral cortex [4], if the greater noise evident with graded firing rate distributions is taken into account.

These effects were found even when the firing rates and the sparseness of the representations were carefully equated across the graded and binary firing rate distribution conditions (e.g. Fig. 5).

The results support the hypothesis that increased noise with the graded firing rate distributions is responsible for the decreased decision or reaction times. Conceptually, one can think that with graded firing rate distributions, a small number of neurons are made more important through their stronger weights and higher firing rates, noting that the variance of a Poisson process is equal to its mean. The influence of the few most highly firing neurons through their particularly strong synaptic weights on other neurons will have the effect of increasing the statistical fluctuations, which will be dominated by the relatively small number of highly firing neurons, and their possibly strong effects on a few other neurons with particularly strong synaptic weights from those highly firing neurons. Effectively the few strongly firing neurons in an attractor with their extra-strong couplings mean that a relatively few neurons dominate the statistical fluctuations, which are large because with the graded firing rate distributions a few neurons have extra high firing rates and extra-strong couplings to each other. In a sense, we can think of the graded firing rate distribution as providing a more sparse representation, with fewer neurons highly active when in a high firing rate attractor state, with the small number of highly active neurons promoting greater statistical fluctuations due to the finite size effect operating with smaller numbers. We note that in an attractor network, prototypical of the design of the neocortex and the hippocampal CA3 region [16], in which the synaptic weights of the recurrent connections are set up by an associative (Hebbian) synaptic modification rule (e.g. equation 2), graded firing rate distributions will always be associated with graded recurrent synaptic weights, and so both can contribute to the effects produced on the noise in the network.

More formally, we can consider the currents injected into a neuron as consisting of a synaptically weighted sum of the input firing rates generated by a Poisson process to each synapse. For a weighted sum of Poisson inputs, the contribution to the variance is more significant from the weight (proportional to its square) than from the rate of the Poisson process (proportional to the value itself). Hence, for two input currents with identical means, with one from the weighted summation of Poisson processes, and the other from the simple summation of Poisson processes, we should expect that the weighted sum in general would have a larger variance. More precisely, let us consider two synaptic inputs 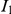 and 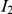


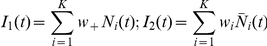
where 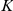 is the number of synapses, 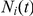 in the binary case is a Poisson process with firing rate 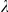 and weight 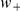, and in the graded rate distribution case 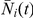 is another Poisson process with firing rate 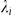 with weight 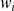. (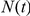 counts the number of spikes in a time interval 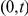. For a Poisson process, 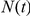 is drawn from a Poisson distribution with parameter 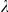.) The means of these two types of input are
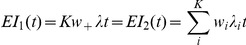
which yields
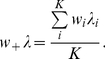
(5)For simplicity, and it is the actual case in our simulations here, we further assume that 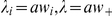, where 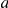 is a positive scaling number. Hence Eq. (5) turns out to be
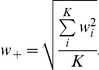
The variances of the two synaptic inputs are
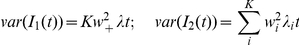
respectively. We can see that in general the second term above, 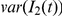, is larger than the first, 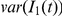, since
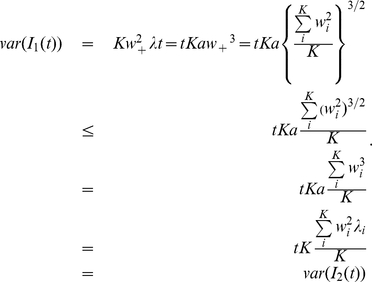
The inequality above is due to Jensen's inequality which states that for any convex function 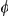, 
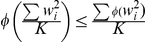
. In our case 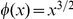. Thus the weighted sum of Poisson processes has greater variance than the sum of Poisson processes when the expected means are equal. Accordingly we would expect more variance of the currents injected into neurons with a graded firing rate and weight distribution than with the binary firing rate and weight distribution when the injected currents are the same. This analysis is supported by our finding that the variance of the NMDA currents injected into each neuron of pools 1 and 2 in the spontaneous period was greater in the graded than the binary rate distribution case (300 vs 254 nA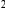, 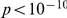), whereas the means were similar (48.3 vs 48.4 nA).

We emphasize that the mean firing rates and mean variances of the decision populations of neurons are very similar for the binary and graded rate distribution cases: it is the distributions that are different, as shown in Fig. 9. The concept here is that for the graded rate distribution representation the subset of neurons with higher than average variance (and firing rates) contribute especially strongly to the noise (i.e. variation, fluctuation) in the system that promotes diffusion [50] across the barrier in the energy landscape (Fig. 1), and that the effect of these neurons is helped by their stronger than average connection weights to other neurons within their decision pool, which enable statistical fluctuations in their rates to be felt especially strongly by the other neurons in the same decision attractor.

To clarify, the descent into the decision attractor basin first has to overcome the energy barrier that keeps the system in the spontaneous stable state (Fig. 1c). Greater variation in the system will mean that this transition is more likely to happen quickly. This is due to the fact that many coincident spikes are needed to overcome this energy barrier. Increased noise means that we are more likely to observe the right set of coincident spikes occurring earlier.

The work described here shows that a potentially useful property of the graded distribution firing rate representations found in the brain [16], [31] is the faster decision times found than with binary firing rate distributions. Given that attractor networks in the cortex have to be large, with thousands of recurrent collateral synapses onto each neuron, as this is the leading factor that determines the number of different memories that can be stored and correctly retrieved [16], [18], [19], the graded firing rate distributions may enable the finite size statistical fluctuations to still influence the processing, and indeed make the processing faster than it would be with binary firing rate distributions. This speed is important, for recurrent collateral processing may be useful at every stage of each sensory hierarchy of cortical processing, yet there may be time for only 20–25 ms of processing at each cortical stage of the hierarchy [16], [55]–[58]. The functions to which the noisy graded firing rate distributions contribute in cortical attractor networks include memory recall, probabilistic decision-making, the facilitation of perceptual detection by stochastic resonance, creative thought, disengagement of attention, and an element of unpredictability of behaviour that can be advantageous [4].

The framework used here can be extended very naturally to account for the probabilistic decisions taken when there are multiple, that is more than two, choices. One such extension models choices between continuous variables in a continuous or line attractor network [59], [60] to account for the responses of lateral intraparietal cortex neurons in a 4-choice random dot motion decision task [61]. In another approach, a network with multiple discrete attractors [62] can account well for the same data. The effects described in the current paper, that the greater spiking-related noise of graded than of binary rate distribution representations can reduce the stability, and increase the speed of decision-making, will apply directly to the discrete attractor scenario, in which greater noise will decrease the escaping time from one state to another in the energy landscape (Fig. 1c) [4].

The graded nature of the firing rate representations in the cortex may of course be adaptive for other reasons than the speed of processing, which might be an added benefit if there are other reasons for graded distribution firing rate representations. If the number of spikes recorded in a fixed time window is taken to be constrained by a fixed maximum rate, one can try to interpret the distribution observed in terms of optimal information transmission [63], by making the additional assumption that the coding is noiseless. An exponential distribution, which maximizes entropy (and hence information transmission for noiseless codes) is the most efficient in terms of energy consumption if its mean takes an optimal value that is a decreasing function of the relative metabolic cost of emitting a spike [64]. This argument would favour sparser coding schemes the more energy expensive neuronal firing is (relative to rest). Although the tail of actual firing rate distributions is often approximately exponential [21], [22], [31], the maximum entropy argument cannot apply as such, because noise is present and the noise level varies as a function of the rate, which makes entropy maximization different from information maximization. Moreover, a mode at low but non-zero rate, which is often observed [16], [21], [31] is inconsistent with the energy efficiency hypothesis.

In conclusion, we have investigated the effects of graded distribution firing rate patterns in a recurrent spiking neural network attractor model of decision-making. The graded rate distributions for the patterns we produced in the numerical simulations took a similar form to those found neurophysiologically. The main finding is that the transition time to an attractor state, or reaction time, is decreased when neurons fire with the more biologically realistic graded firing rates across the neuronal populations. One advantage of these graded firing rate representations is that they provide a sparse distributed representation with independence of the information provided by each neuron, allowing for the useful properties in associative networks of generalization, completion, and graceful degradation [16], [37]. It has been argued elsewhere [64] that graded distribution firing rates may also maximize information transmission for a given mean rate of firing, and therefore energy consumption, given that high average firing rates require more metabolic expenditure. [However, an alternative account of the graded distributions is that they arise with integrate-and-fire neurons with slow fluctuations in the inputs (reflecting different stimuli) and fast fluctuations in the inputs (reflecting for example trial-by-trial variability in the response to a given stimulus, to which the effects of the spiking-related, close to Poisson, high entropy, fluctuations in the number of spikes in a short time window analyzed in this paper could contribute) [30]. The long tail of graded firing rate probability distributions may also be required for cost efficiency [65].] The results described here show that an additional useful property of the graded representations found in the brain is that they may increase the speed of decisions, reducing the time required for many processes such as memory recall as well as more conventionally understood decision-making [4]. Given that cortical computation frequently requires a hierarchical series of cortical stages in each of which attractor processes may contribute, the cumulative effect on the increased speed of processing of graded firing rate representations over a series of cortical stages may be considerable [16], [57].

We emphasize that it is important to understand the effects of noise in networks in the brain, and its implications for the stability of neuronal networks in the brain. For example, a stochastic neurodynamical approach to schizophrenia holds that there is less stability of cortical attractor networks involved in short-term memory and attention due to reduced functioning of the glutamate system, which decreases the firing rates of neurons in the prefrontal cortex, and therefore, given the spiking-related noise that is present, the depth of the basins of attraction. This it is suggested contributes to the cognitive changes in schizophrenia, which include impaired short-term memory and attention [32], [33], [35]. In another example, a stochastic neurodynamical approach to obsessive compulsive disorder holds that there is overstability in some networks in prefrontal cortex and connected areas due to hyperglutamatergia [34], [36]. In both these cases, and also in normal brain function in relation to decision-making, memory recall, etc, it is important to know to what extent noise contributed by randomness in the spiking times of individual neurons for a given mean rate contributes to stochastic effects found in the brain which affect decision-making, stability, and which may if the stability is disturbed contribute to neuropsychiatric disorders. In this context, the findings described in this paper are important for understanding normal and disordered brain function. In particular, a very interesting implication of the findings described here is that there is more noise with the graded rate distribution representations found in the brain (see [16] Appendix 3 on information encoding in the brain) than with binary firing rate distributions (which are often used in simulations, because they are amenable to mean-field analyses [1], [2]). Thus when noise is found to be a significant factor in the operation of integrate-and-fire decision-making networks with binary firing rates up to sizes that have been tested of 4096 neurons each with 4096 synapses per neuron, then it is likely that with graded firing rates, spiking-related noise will continue to be a factor in the operation of cortical circuitry even up to the larger numbers of recurrent collateral synapses onto each neuron. For example, in the cerebral cortex there are typically in the order of 9,000 recurrent collateral synapses onto onto each cortical pyramidal cell, from a total of in the order of 18,000 synapses [13], [16].

## Supporting Information

Material S1 Supporting material.
(PDF)
