# Supplementary material for: Noise in Attractor Networks in the Brain Produced by Graded Firing Rate Representations
Source: PLoS One. 2011 Sep 8;6(9):e23630. doi: 10.1371/journal.pone.0023630 (PMC3169549; doi:10.1371/journal.pone.0023630)
Supplement: Material S1 — Supporting material. (PDF) [file pone.0023630.s001.pdf]

Supplementary Material S1: Noise in attractor networks in the brain produced by graded firing rate representations

Tristan J. Webb, University of Warwick

Department of Computer Science and Complexity Science Centre, Coventry CV4 7AL, UK

Edmund T. Rolls, Oxford Centre for Computational Neuroscience, Oxford, UK*

and University of Warwick, Department of Computer Science, Coventry CV4 7AL, UK

Gustavo Deco, Universitat Pompeu Fabra, Theoretical and Computational Neuroscience

Roc Boronat 138, 08018 Barcelona, Spain

and

Jianfeng Feng, University of Warwick, Department of Computer Science, Coventry CV4 7AL, UK

July 30, 2011

*Corresponding author. Oxford Centre for Computational Neuroscience, Oxford, UK. Email: Edmund.Rolls@oxcns.org, url: http://www.oxcns.org

This Supplementary Material S1 summarizes the parameters used in the simulations shown in the paper and provides a tabular description of the network following the prescription of (Nordlie, Gewaltig & Plesser 2009).

**A**	**Model Summary**
**Populations**	Two: excitatory, inhibitory
**Topology**	—
**Connectivity**	Fully connected
**Neuron model**	Leaky integrate-and-fire, fixed threshold, fixed refractory period, NMDA
**Channel models**	—
**Synapse model**	Instantaneous jump and exponential decay for AMPA and GABA and exponential jump and decay for NMDA receptors
**Plasticity**	Synaptic facilitation
**Input**	Independent fixed-rate Poisson spike trains to each selective population
**Measurements**	Spike activity

**B**	**Populations**	
Total number of neurons	$N = 500$	
Excitatory neurons	$N_E = 0.8 * N$	Neurons in each selective pool $N_{selective} = N_E \cdot sparseness$
Inhibitory neurons	$N_I = 0.2 * N$	

C	Neuron and Synapse Model
**Type**	Leaky integrate-and-fire, conductance-based synapses
**Subthreshold dynamics**	$$C_m \frac{dV(t)}{dt} = -g_m(V(t) - V_L) - I_{\text{syn}}(t),$$ $$I_{\text{syn}}(t) = I_{\text{AMPA,ext}}(t) + I_{\text{AMPA,rec}}(t) + I_{\text{NMDA}}(t) + I_{\text{GABA}}(t)$$
**Spiking**	If $V(t) > V_\theta \wedge t > t^* + \tau_{rp}$<br>1. set $t^* = t$<br>2. emit spike with time-stamp t^*<br>3. $V(t) = V_{reset}$
**Synaptic currents**	$$I_{\text{AMPA,ext}}(t) = g_{\text{AMPA,ext}}(V(t) - V_E) \sum_{j=1}^{N_{\text{ext}}} s_j^{\text{AMPA,ext}}(t)$$ $$I_{\text{AMPA,rec}}(t) = g_{\text{AMPA,rec}}(V(t) - V_E) \sum_{j=1}^{N_E} w_j s_j^{\text{AMPA,rec}}(t) u_j(t)$$ $$I_{\text{NMDA}}(t) = \frac{g_{\text{NMDA}}(V(t) - V_E)}{1 + \gamma \exp(-\beta V(t))} \sum_{j=1}^{N_E} w_j s_j^{\text{NMDA}}(t) u_j(t)$$ $$I_{\text{GABA}}(t) = g_{\text{GABA}}(V(t) - V_I) \sum_{j=1}^{N_I} s_j^{\text{GABA}}(t)$$
**Fraction of open channels**	$$\frac{ds_j^{\text{AMPA,ext}}(t)}{dt} = -s_j^{\text{AMPA,ext}}(t)/\tau_{\text{AMPA}} + \sum_k \delta(t - t_j^k - \delta)$$ $$\frac{ds_j^{\text{AMPA,rec}}(t)}{dt} = -s_j^{\text{AMPA,rec}}(t)/\tau_{\text{AMPA}} + \sum_k \delta(t - t_j^k)$$ $$\frac{ds_j^{\text{NMDA}}(t)}{dt} = -s_j^{\text{NMDA}}(t)/\tau_{\text{NMDA,decay}} + \alpha\, x_j(t)(1 - s_j^{\text{NMDA}}(t))$$ $$\frac{dx_j(t)}{dt} = -x_j(t)/\tau_{\text{NMDA,rise}} + \sum_k \delta(t - t_j^k - \delta)$$ $$\frac{ds_j^{\text{GABA}}(t)}{dt} = -s_j^{\text{GABA}}(t)/\tau_{\text{GABA}} + \sum_k \delta(t - t_j^k - \delta),$$

D	Input
**Type**	**Description**
Poisson generators	Fixed rate N_{ext} synapses per neuron, with each synapse driven by a Poisson process

E Measurements
Spike activity

Table 1: Parameters used in the integrate-and-fire simulations

C_{m} (excitatory)	0.5 nF
C_{m} (inhibitory)	0.2 nF
g_{m} (excitatory)	25 nS
g_{m} (inhibitory)	20 nS
V_{L}	-70 mV
V_{thr}	-50 mV
V_{reset}	-55 mV
V_{E}	0 mV
V_{I}	-70 mV
$g_{\mathrm{AMPA,ext}}$ (excitatory)	2.08 nS
$g_{\mathrm{AMPA,rec}}$ (excitatory)	0.208 nS
g_{NMDA} (excitatory)	0.654 nS
g_{GABA} (excitatory)	2.5 nS
$g_{\mathrm{AMPA,ext}}$ (inhibitory)	1.62 nS
$g_{\mathrm{AMPA,rec}}$ (inhibitory)	0.162 nS
g_{NMDA} (inhibitory)	0.516 nS
g_{GABA} (inhibitory)	1.946 nS
$\tau_{\mathrm{NMDA,decay}}$	100 ms
$\tau_{\mathrm{NMDA,rise}}$	2 ms
τ_{AMPA}	2 ms
τ_{GABA}	10 ms
τ_{rp}(excitatory)	2 ms
τ_{rp}(inhibitory)	1 ms
α	0.5 ms^{-1}
γ	$[\mathrm{Mg}^{2+}]/(3.57\mathrm{mM}) = 0.280$
β	$0.062\,\mathrm{mV}^{-1}$
sparseness, a	0.10
N_{ext}	800

Table 2: Connection parameters used in the model

w_+	2.1
w_-	0.877

References

Nordlie, E., Gewaltig, M. O. & Plesser, H. E. (2009). Towards reproducible descriptions of neuronal network models, *PLoS Computational Biology* **5**: e1000456.
